# D-dimer for the exclusion of cerebral venous thrombosis: a meta-analysis of low risk patients with isolated headache

**DOI:** 10.1186/s12883-015-0389-y

**Published:** 2015-07-28

**Authors:** Imanda M.E. Alons, Korné Jellema, Marieke J.H. Wermer, Ale Algra

**Affiliations:** Department of Neurology, MCH Westeinde, Lijnbaan 32, 2501 CK The Hague, The Netherlands; Department of Neurology, LUMC, Albinusdreef 2, 2333 ZA Leiden, The Netherlands; Department of Clinical Epidemiology, LUMC, Albinusdreef 2, 2333 ZA Leiden, The Netherlands; Department of Neurology and Neurosurgery, Brain Center Rudolph Magnus, UMC, Utrecht, The Netherlands; Julius Center for Health Sciences and Patient Care, UMC, Utrecht, The Netherlands

**Keywords:** Cerebral sinus thrombosis, D-dimer, Isolated headache, Diagnostic marker, Meta-analysis

## Abstract

**Background:**

Patients with isolated headache may have cerebral venous thrombosis (CVT). D-dimers are proven sensitive in excluding deep venous thrombosis (DVT) and pulmonary embolism (PE) in low risk patients. We aimed to determine whether D-dimer may play the same role in low risk CVT patients with isolated headache.

**Methods:**

We included consecutive patients suspected of CVT from our teaching hospital with isolated headache, a normal neurological examination and normal standard head CT in whom D-dimer was determined. Additionally we did a systematic review on articles describing consecutive patients suspected of CVT with isolated headache and their D-dimer values. CVT was investigated with CT or MR venography in all patients.

**Results:**

A total of 636 consecutive patients were collected from our own data and the literature search. Of 45 CVT patients one had a negative D-dimer (7.5 %). Sensitivity of D-dimer for diagnosing CVT was 97.8 % (95 % CI: 88.2–99.6 %), specificity was 84.9 % (95 % CI: 81.8–87.7 %), positive predictive value was 33.1 % (95 % CI: 25.2–41.7 %), negative predictive value was 99.8 % (95 % CI: 98.9–100 %). Another 56 isolated headache CVT patients were identified in literature, lacking consecutive isolated headache controls. Sensitivity of D-dimer for diagnosing CVT including these patients was 87.1 % (95 % CI: 79.0–93.0 %).

**Conclusions:**

D-dimers have a high negative predictive value in patients with isolated headache for excluding CVT. Sensitivity is lower but comparable to the values accepted in PE and DVT. Low risk patients were defined as headache patients with a normal neurological examination, normal standard head CT and absence of risk factors such as pregnancy or puerperium. Normal D-dimers in these patients may reduce unnecessary imaging, making it a potential valuable marker.

## Background

Headache frequently leads to emergency room consultation. It is vital to exclude secondary forms of headache requiring further treatment such as cerebral venous thrombosis (CVT). CVT is accompanied by headache in 89 % of cases [1, 2]. It may present with headache alone in 14 % [3] and acute headache in 3–13 % of cases [4–6]. Patients with isolated headache seem to have a good prognosis, however, CVT patients with isolated headache who present early (<7 days) are more at risk to deteriorate neurologically than patients with isolated headache who present later, making diagnosis in an early stage important [6].

D-dimers have been proven useful in the diagnosis of pulmonary embolism (PE) and deep venous thrombosis (DVT) [7, 8]. Clinical factors are translated to the Wells score, ranking patients to high or low risk categories [7]. The Wells score for PE is calculated based on clinical parameters and patient history. The score includes clinical signs of DVT, PE being the most likely diagnosis, heart rate over 100 bpm, recent immobility or surgery, hemoptysis, previous PE or DVT or the presence of malignancy, Each item in the Wells score has a rating and patients with 1.5 point or less are rated low risk for PE. When low risk Wells score patients have a normal D-dimer, the post-test probability of DVT or PE is 0.5–2 % when using a sensitive ELISA quantative assay [9]. In these low risk patients it is safe to forego further imaging such as echo venography of the lower limbs or pulmonary spiral CT. Sensitivity of D-dimer varies from 83–96 % in diagnosing DVT patients and 75–97 % in diagnosing PE patients, depending on the performed D-dimer test [10].

Whether the measurement of D-dimer can play a similar role in suspected cerebral vein thrombosis remains uncertain. A recent meta-analysis of the literature concluded that D-dimer may be useful as a diagnostic tool in CVT patients in general, but an analysis for isolated headache was not the aim of this review [11]. According to American guidelines patients with a high clinical suspicion of CVT should receive neuro-imaging regardless of D-dimer results [12]. This makes D-dimer a superfluous test in high risk cases. The definition of low risk patients for CVT requires different criteria from the Wells score for PE of DVT. In current practice the presence of neurological deficit and abnormalities on standard head CT, but also known risk factors for CVT such as pregnancy or use of oral contraceptives are weighed in the decision to perform further diagnostic work-up. Whether D-dimers are sensitive enough to exclude CVT in low risk patients, without these characteristics and isolated headache is unknown. In these patients D-dimer would be valuable to decide whether or not additional imaging is necessary. There are conflicting and limited data on patients with isolated headache. One often cited study found negative D-dimers in five out of 19 (26 %) patients with CVT and isolated headache [13]. Another prospective study found no false negative D-dimers in 20 patients with isolated headache [14].

We aimed to assess whether D-dimer is a valuable test in CVT patients with isolated headache and a low risk of CVT.

## Methods

### Patient study

We retrospectively included all consecutive patients with headache who presented to the emergency room of our large teaching hospital from January 2010 to December 2014 with headache if a D-dimer was determined at presentation and the presence of CVT was examined with CT venography or MRI or both. D-dimers were determined routinely by the treating physician when CVT was suspected. As this study was done retrospectively reviewing patient charts, formal approval of the local ethics committee was not applicable. We defined isolated headache as headache in the absence of abnormalities at neurological examination: lowered consciousness, seizures, focal motor deficit, focal sensory deficit, visual field defects, abnormal pupillary responses, eye movement disorders, papillary edema and pathological tendon or plantar reflexes. We only included patients with normal standard head CT. We felt that in the presence of hemorrhage or other abnormalities suspected for CVT at standard CT, follow up diagnostics would have been done regardless of D-dimer values. We included patients with known risk factors for CVT including oral contraceptive use, pregnancy or in puerperium.

We recorded headache characteristics including duration and onset, the presence of nausea and vomiting and also the number of affected sinuses. Also diagnoses at presentation and after 1 year of follow-up were recorded.

D-dimer was determined by the in house laboratory with a Roche second generation latex essay, turbidimetric method on a modular chemistry analyzer. D-dimer was deemed negative if lower than 0.5 μg/ml. We adopted this cut-off value for CVT also as data differently to this are lacking.

Patients underwent scanning by GE Lightspeed 64 slice CT scanner using intravascular iodine contrast. The CT angiography depicted both the arterial and venous system of intra- and extracranial vessels, by scanning the early and late phase of contrast passage. MRA was done with a 1.5 Tesla Siemens MRI with gadolinium enhanced venography or Time of Flight (TOF) imaging.

### Literature search

Literature search for articles was done in Pubmed, and Embase with the following search criteria: “cerebral venous thrombosis”, “cerebral venous sinus thrombosis”, “Ddimer”, “D-dimer”, “isolated headache” and combinations of these. We also scanned reference lists of found articles for possible inclusions. We included original articles focusing on D-dimer determination in consecutive patients with suspected CVT, where the presence of isolated headache could be determined. Articles were excluded for the meta-analysis if they were reviews or comments. Articles were scanned for information on patients with isolated headache. If insufficient data were given in the articles, authors were approached for unpublished data on these patients. We evaluated the articles with the QUADAS-2 checklist, which is recommended to evaluate the risk of bias and applicability of primary diagnostic accuracy studies. [15]

In these articles data on duration of complaints, risk factors for CVT and headache characteristics were also recorded.

### Data analysis

Data were evaluated with 2×2 contingency tables and we calculated 95 % confidence intervals for proportions. Sensitivity, specificity and the negative and positive predictive value could be calculated for both our own data and the data extracted from the systematic review. An additional calculation of sensitivity was done including patients from literature with established CVT that either lacked isolated headache controls or with insufficient information on the control group given in the article. As a matched control group was not present for these patients it was not possible to calculate specificity, negative predictive value or positive predictive value for this entire group.

## Results

### Patient study

In our center we found 672 patients who presented to the emergency room with headache in whom D-dimer was determined. Of these patients 312 received sufficient imaging to investigate the presence of CVT and 149 of these (47.7 %) had a normal neurological examination and normal standard head CT. Neuro-imaging in these patients consisted of CTA/V in 105, MRI in 23 and both modalities in 18 patients (Table 1). Average age was 42 years and there were 42 men (28 %). Three patients had CVT (2.7 %) and all had a raised D-dimer. Of the 146 patients without CVT, 63 had a raised D-dimer. Sensitivity was 100 % (95 % CI: 30.5–100 %), specificity 56.8 % (95 % CI: 48.4–65.0 %), positive predictive value was 4.55 % (95 % CI: 1.00–12.7 %) and negative predictive value 100 % (95 % CI: 95.6–100 %). Average time between symptom onset and diagnosis was 11 days (SD 16) in the CVT group and 16 days (SD 88) in the non CVT group. Two patients were pregnant and eight had a known use of oral contraceptives. Two patients using oral contraceptives had CVT and both had a raised D-dimer.

**Table 1:** Characteristics of included patients from our hospital

	CVT +	CVT -	Total
Patients	3	146	149
Age (mean)	35	42	42
Men (%)	3 (100 %)	39 (27 %)	42 (28 %)
Side of headache			
Bilateral	3 (100 %)	105 (71.9 %)	108 (72.5 %)
Unilateral	0	36 (24.7 %)	36 (24.1 %)
Local(eye)	0	5 (3.4 %)	5 (3.4 %)
Acute onset of headache	0	62 (41.6 %)	62 (41.6 %)
Days to presentation median (IQR)	2 (0–8)	3 (1–7)	3 (1–7)
Nausea	3 (100 %)	81 (55.5 %)	84 (56.4 %)
Vomiting	3 (100 %)	51 (34.9 %)	54 (36.2 %)
CTA/V	0	105 (72 %)	105 (70.5 %)
MRA/V	0	23 (16 %)	23 (15.5 %)
Both CTA/V en MRA/V	3 (100 %)	18 (12 %)	21 (14 %)
Raised D-dimer	3 (100 %)	63 (43 %)	66 (44 %)
Diagnosis			
Tension type headache		81	
Migraine		24	
Para Infectious		8	
Paroxysmal hemicrania		4	
SAH		5	
IIH		2	
Other		22	

*CTA CT* angiography*, CTV CT* venography*, MRA MR* angiography*, SAH* Subarachnoid hemorrhage*, IIH* idiopathic intracranial hypertension

### Literature search

**Fig. 1:**
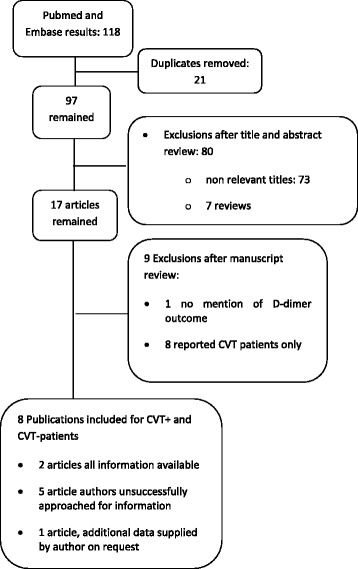
Literature search results for articles on patients with isolated headache, with and without CVT and D-dimer determination

**Fig. 2:**
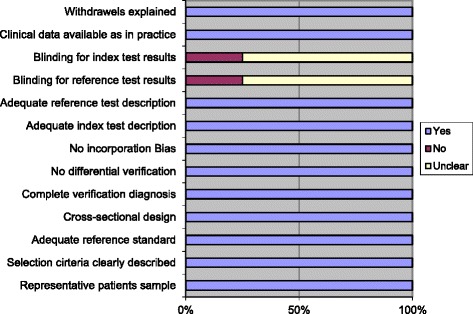
QUADAS-2 Checklist of three included articles and own methods

We identified 118 potentially relevant articles. After excluding duplicates, scanning of titles and abstracts 17 articles remained (see Fig. 1). After further evaluation of these articles we found eight articles that described both CVT and non CVT groups with isolated headache and their D-dimer levels (Table 2). Full information could be immediately extracted from two articles [14, 16]. In total six authors were approached for additional information. We received additional information from one of the approached authors adding information on 173 patients [17]. Characteristics of the included articles were evaluated with the QUADAS-2 (Fig. 2).

**Table 2:** Study characteristics of studies describing consecutive patients suspected of CVT

	Study design	All/isolated headache patients	D-dimer test	Cut-off value	Imaging	Missing information
Tardy et al. [16]	Prospective	52/23	ELISA Vidas assay	>0.5 μg/ml	MRA	None missing
Kosinski, et al. [14]	Prospective	343/291	2nd generation latexagglutination	>0.5 μg/ml	MRA or CTV	None missing
Meng, et al. [17]	Prospective	233/173	Immuno- turbidimetric assay	>0.5	MRI/MRV	None missing
Tanislav et al. [23]	Retrospective	239/X	Turbidimetric	>0.19 μg/ml	CTV or MRV	Isolated headache unclear
Ghaffarpour et al. [24]	Prospective	104/X	Conventional ELISA	>0.5 μg/ml		Isolated headache unclear
Gouda, Sabry [21]	Prospective	104/X	2nd generation latexagglutination	>0.5 μg/ml	TOF MRA	isolated headache unclear in non CVT group
Lalive, et al. [20]	Prospective	54/X	ELISA Vidas assay	>0.5 μg/ml	MRA	Isolated headache unclear in non CVT group
Cucchiara, et al. [22]	Prospective	31/X	ELISA Vidas assay	>0.5 μg/ml	MRV	Isolated headache unclear in non CVT group

*MRA MR* angiography*, MRV MR* venography*, CTV CT* venography*, TOF* time of flight*, CVT* cerebral venous thrombosis

We included 487 patients from the three articles with complete information [14, 16, 17]. Of these patients 42 had CVT (8.6 %) of whom one (2.4 %) had a negative D-dimer. One patient was pregnant and 14 used oral contraceptives. This information was only given for patients with established CVT. All patients with these risk factors had a positive D-dimer. There were 445 patients without CVT, of whom 416 had a negative D-dimer.

### Combined data

**Fig. 3:**
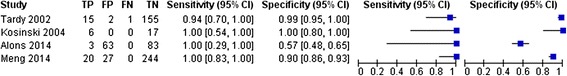
Overview of the sensitivity and specificity of the included articles

We included 636 consecutive patients with suspected CVT and normal neurological examination from both our data and the collected articles (Table 3). In this group 45 had CVT (7.1 %) of whom 1 had a negative D-dimer (1.6 %). Sensitivity was 97.8 % (95 % CI: 88.2–99.6 %), specificity was 84.9 % (95 % CI: 81.8–87.7 %), positive predictive value was 33.1 % (95 % CI: 25.2–41.7 %), negative predictive value was 99.8 % (95 % CI: 98.9–100 %) (Fig. 3). After D-dimer determination there is a 0.2 % chance of CVT in patients with isolated headache and a negative D-dimer. The prevalence of CVT in this group with isolated headache was 7.5 %. There is a 6.9 % post-test reduction of the chance of having CVT in patients with normal neurological examination after D-dimer determination.

**Table 3:** Data on 636 patients with isolated headache

	CVT		No CVT	
	*D*-*dimer raised*	*D*-*dimer normal*	*D*-*dimer raised*	*D*-*dimer normal*
Tardy et al., 2002	6	0	0	17
Kosinski et al., 2004	20	0	27	244
Meng et al. 2014	15	1	2	155
Alons et al., 2015	3	0	63	83
Total	44	1	92	499

We also evaluated the articles that included only patients with established CVT [13, 18, 19] and articles with insufficient data on non-CVT patients [20–22]. From these articles we included another 56 CVT patients (Table 4). Of the combined 101 patients with CVT and isolated headache 13 (12.9 %) had a false negative D-dimer. D-dimer in this group had a sensitivity of 87.1 % (95 % CI: 79.0–93.0 %).

**Table 4:** D-dimer results of CVT patients from articles describing established CVT patients with insufficient data on isolated headache in non CVT group

	D-dimer raised	D-dimer normal	Total (% D-dimer -)
Misra et al., 2009	2	1	3 (33 %)
Hiltunen et al., 2013	17	2	19 (10 %)
Crassard et al., 2005	14	5	19 (26 %)
Gouda, Sabry, 2010	3	3	6 (50 %)
Lalive et al., 2003	5	1	6 (17 %)
Cucchiara et al., 2005	3	0	3 (0 %)
Total	44	12	56 (27 %)

In only 33 of the 101 CVT patients information on sinus involvement was available [13, 16, 18, 20–22]. Of these patients 13 had a single affected sinus and D-dimer was negative in 7 cases (54 %). In 11 patients with two affected sinuses D-dimer was negative in four (36 %). In nine patients with three or more affected sinuses none of D-dimers were negative (0 %). Information on duration of symptoms was available for 42 of 101 CVT patients [16, 17, 20–23]. Seventeen patients had complaints <7 days and 6 (35 %) of these patients had negative D-dimer. Of the 18 Patients with headache >7 days 5 (28 %) had negative D-dimer. Of the seven patients with headache >14 days 3 (43 %) had negative D-dimer.

Of the 25 patients who were either pregnant, in puerperium or using oral contraceptives, 17 had CVT and none had a false negative D-dimer. However data on these risk factors for non-CVT controls were not given or could not be reliably deduced in the patients from literature.

## Discussion

Our study suggests that D-dimer is a sensitive diagnostic tool in excluding CVT in low risk patients. We found a high negative predictive value in patients with isolated headache suspected of CVT in our meta-analysis that combined data from our own and three previous series. This is comparable with the values found in PE and DVT when a low risk Wells score, based on physical examination, is combined with negative D-dimer. Our meta-analysis describes the largest group of patients with isolated headache studied for the diagnostic value of D-dimer in recent literature. This is also the only study focusing on low risk CVT patients, making the results valuable for everyday practice.

Our study has limitations. First, insufficient data on isolated headache and concomitant D-dimer levels were available from six potential useful articles [13, 17–20, 22]. We attempted to obtain additional information from the concerned authors but received information from only one [17]. However, we did collect data on a large number of patients allowing a sufficiently precise estimation of the diagnostic value of D-dimer in this low risk group. Second, we could not determine how many headache patients presented to our hospital in whom D-dimer was not determined in this period. Unfortunately, it is not possible to reliably retrieve this information from the hospital information system. This means that in our evaluation very low risk patients may have been missed compromising the generalizability of the findings. On the other hand, the addition of patients from studies performed in other large hospitals in several countries improved the generalizability of our results..

Finally, the D-dimer determination method varied over the cited articles; however, most articles used quantitative latex assays, with high sensitivity with the same cut-off value. It was not possible to calculate D-dimer cut-off levels in this group as exact values were not given in most articles, in particular not in patients without CVT.

We defined patients to have a low risk of CVT when they had a normal neurological examination and normal standard head CT. The definition of a normal neurological examination might be a point of discussion as papillary edema for instance may be difficult to determine reliably and in many cases is not judged by the treating physician at all. In an earlier study patients with papillary edema were included in a cohort with isolated headache [6]. In our group two patients with papillary edema alone had a negative D-dimer, and were excluded from our group of patients with isolated headache. Also a symptom like tinnitus which may be present in raised intra cranial pressure is often not mentioned and could not be included in our definition.

A raised risk of CVT exists during pregnancy and puerperium and in patients using oral contraceptives, but also in older patients and patients with underlying malignancy. These patients are also at risk of having a false positive D-dimer. In the entire group three patients were reported to be pregnant and 22 were reported to be using contraceptives. However, the use of contraceptives or presence of pregnancy in the patients from literature could only be clearly deduced in those with established CVT. The limited number of patients and insufficient reporting of pregnancy and use of contraceptives makes it difficult to give recommendations. Caution in this high risk group seems warranted and we feel these patients should undergo follow up neuro-imaging when CVT is suspected regardless of D-dimer outcome. Of all consecutive patients with CVT there were 1.6 % false negative D-dimers. Earlier concerns regarding usability of D-dimer in this patient group was based on percentages found in studies citing percentages of false negative D-dimers ranging from 10–50 % [12, 15, 18, 20–22]. However these articles did not mention D-dimer results in non-CVT patients or gave insufficient data on this control group. In our own patient group no false negative D-dimers were found, which was confirmed by three studies, including two large prospective studies [11, 17, 23]. Including these articles in our meta-analysis, the sensitivity of D-dimer in isolated headache patients for detection of CVT is 87 % (95 % CI: 79.0–93.0 %). This sensitivity is lower, but comparable to, sensitivity found in various tests used for D-dimer determination in PE and DVT patients [10]. Unfortunately as no matched isolated headache controls are described, the calculation of specificity and the negative and positive predictive value for this entire group is impossible. As the patients in most of these articles were collected over time in tertiary headache referral centers, they may cause an overestimation of CVT, with large numbers of controls balancing them out.

When evaluating the risk for false negative D-dimer this is higher in patients with a single affected sinus and patients with a longer duration of complaints [14]. Unfortunately information on both these parameters was only available in a small number of patients and conclusions on these factors cannot be drawn from our data. These parameters are an important focus of future prospective research.

In patients with isolated headache D-dimer could become a useful screening tool to exclude CVT and may help reduce unnecessary additional imaging using intra-venous contrast or MRI. In the presence of risk factors for CVT such as pregnancy, puerperium, use of oral contraceptives, neurological symptoms and abnormalities on standard head CT it remains necessary to perform diagnostic follow-up. As is the case in DVT and PE D-dimers cannot be seen a stand-alone test and must be combined with clinical risk factors. The high negative predictive value of 99.8 % may aid the physician in decision making, although a positive result does not prove the presence of CVT due to low positive predictive value. Although sensitivity is lower, it is comparable to sensitivity accepted in excluding PE and DVT. In this series the use of negative D-dimer would have avoided additional neuro-imaging for diagnosing CVT in 502 patients, whereas only 89 patients with false positive D-dimer would have been scanned unnecessarily. One patient would have been missed. Prospective studies confirming our findings concerning low risk CVT patients are needed.

## Conclusion

D-dimers have a high negative predictive value in patients with isolated headache for excluding CVT. Sensitivity is lower but comparable to the values accepted in PE and DVT. Low risk patients were defined as headache patients with a normal neurological examination, normal standard head CT and absence of risk factors such as pregnancy or puerperium. Normal D-dimers in these patients may reduce unnecessary imaging, making it a potential valuable marker.

## Abbreviations

CVT: Cerebral venous thrombosis
DVT: Deep venous thrombosis
PE: Pulmonary embolism
CT: Computer tomography
MRI: Magnetic resonance imaging
